# Evaluation of *Mycoplasma mycoides* subsp. *mycoides* antigens capable of stimulating host IRG-47 release identifies Mmm604, Mmm605, and Mmm606 as potential subunit vaccine antigens

**DOI:** 10.1128/iai.00186-25

**Published:** 2025-09-09

**Authors:** Tong Liu, Huanjun Zhao, Qi Wu, Yukun Wei, Jiuqing Xin, Qiao Pan

**Affiliations:** 1 National Contagious Bovine Pleuropneumonia Reference Laboratory, State Key Laboratory for Animal Disease Control and Prevention, Harbin Veterinary Research Institute, Chinese Academy of Agricultural Sciences 687216 , Harbin, China; Tulane University, New Orleans, Louisiana, USA

**Keywords:** potential subunit vaccine antigens, cell-mediated immune response, IFN-γ-inducible protein 47, vaccine, contagious bovine pleuropneumonia

## Abstract

Contagious bovine pleuropneumonia (CBPP), caused by *Mycoplasma mycoides* subsp. *mycoides* (Mmm), is a devastating cattle disease with high morbidity and mortality, threatening cattle productivity in Sub-Saharan Africa and potentially in parts of Asia. Cross-border livestock trade increases the risk of CBPP introduction or reintroduction. Current vaccines were developed from attenuated Mmm strains in the last century and face limitations regarding animal welfare, immunity duration, and adverse reactions, necessitating new vaccine strategies. Subunit vaccines offer a promising alternative, but identifying effective antigens is critical. Given the key role of cellular immunity in CBPP control, we focused on antigen identification that elicits a host cellular immune response. This study explores antigen candidates based on Ben-181, a vaccine that successfully eradicated CBPP in China. Ben-181 specifically induces interferon-γ (IFN-γ)-dependent IRG-47 expression, and IFN-γ correlates with cellular immune responses. We propose IRG-47 as a potential marker for Mmm antigen screening. Comparative genomic analysis between Ben-181 and the non-immunoprotective strain Ben-468 identified 35 proteins potentially linked to IRG-47 expression. Further screening revealed Mmm604, Mmm605, and Mmm606 as inducers of IRG-47 release. Intranasal immunization with these proteins in mice enhanced splenic lymphocyte proliferation, CD8 +T cell activation, a mixed Th1/Th2/Th17 response, and humoral antibody production. Mmm604 and Mmm606 also trigger mucosal antibody responses in mice. These proteins effectively stimulate cellular and humoral responses, making them promising candidates for Mmm subunit vaccine development. Our study highlights the potential of IRG-47 in Mmm antigen screening.

## INTRODUCTION

Contagious bovine pleuropneumonia (CBPP) is a severe respiratory infectious disease of *Bovidae* caused by *Mycoplasma mycoides* subsp. *mycoides* (Mmm) (1). Mmm belongs to the genus *Mycoplasma*, a large group of the smallest, wall-less bacteria capable of self-replication. Mmm induces substantial pathologic alterations in cattle respiratory tracts and lungs (2, 3). CBPP outbreaks can cause mortality rates exceeding 50%, reaching up to 90% in severe cases, severely impacting animal welfare and livestock production (4, 5). It is one of the six priority diseases officially recognized by the World Organization for Animal Health (WOAH, https://www.woah.org/en/what-we-do/animal-health-and-welfare/official-disease-status/) and the only non-viral disease among them. Reports of CBPP date back to the 16th century in Europe, with global spread beginning in the 19th century (6). While many countries have eradicated CBPP through culling or post-immunization eradication strategies, it remains endemic in Sub-Saharan Africa, causing an estimated €44.8 million in annual losses (2, 7, 8). Additionally, due to insufficient monitoring and reporting, the prevalence of CBPP in several Asian countries remains unknown (2).

CBPP has been widely prevalent in China since the early 20th century, resulting in significant economic losses. Since the 1960s, China began using an attenuated vaccine developed through successive passages of the highly virulent isolate Ben-1 in heterologous animals and successfully controlled and eradicated domestic CBPP (4). This vaccine provides 95%–100% protection over 2 years, which is superior to other mycoplasma vaccines and most commercial ruminant vaccines. However, its production method (intraperitoneal inoculation of sheep) no longer meets modern animal welfare standards (9). Currently available attenuated vaccines T1/SR and T1/44, prepared from Tanzanian T1 strains, also face limitations in terms of a short period of protection and the production of severe adverse reactions (2). Therefore, new or improved CBPP vaccines are urgently required to control the disease epidemics/re-epidemics. Subunit vaccines, known for inducing strong mucosal, humoral, and cellular immune responses at a lower cost, have emerged as a promising direction for CBPP vaccine development.

Vaccines’ effectiveness against many infectious diseases is largely dependent on serum antibodies and the enhancement of cell-mediated and local immunity (10). Antibody levels detected by serological tests have always been poor indicators of protection and often linked to post-challenge pathological reactions. Evidence suggests that animals with CBPP sequestra exhibit higher antibody levels against surface proteins than those that cleared Mmm from their lungs. High antibody titers might contribute to pathological changes like vasculitis (11). In contrast, cell-mediated immune responses are generally considered more crucial for protection against *Mycoplasma*-induced pneumonia (12). Interferon-gamma (IFN-γ) is an immunomodulatory cytokine that drives CD4 +T cell differentiation into Th1-type cells and further enhances Th1-type cytokine release, thereby strengthening cellular immune responses (13). Endogenous IFN-γ level is a critical marker of cellular immunity, which is essential for combating Mmm-induced bovine pleuropneumonia (14, 15). Effective vaccine candidates should stimulate IFN-γ release to boosting T lymphocyte activation and Th1 responses (15). Given its important role, IFN-γ release in response to Mmm antigen stimulation may have the potential to serve as a marker for screening candidate antigen to develop subunit vaccines.

The commercial IFN-γ ELISpot assay, while sensitive and specific for detecting cell-produced IFN-γ, is both costly and technically complex. In this study, an immunoprotection assay using the vaccine strain Ben-181 revealed that it specifically stimulated the IFN-γ-inducible GTPase IRG-47 expression in bovine lungs. IRG-47 belongs to the 47 kDa immunity-related GTPase family, whose only known function is to play a key role in attacking the vacuolar membranes of intravacuolar parasites in mice (16). Current research shows that IRG-47 is exclusively expressed under IFN-γ regulation (17). These findings suggest that detecting IRG-47 expression via Western blotting could offer a simpler, cost-effective alternative to IFN-γ ELISpot for identifying IFN-γ-inducing antigens in Mmm subunit vaccine development. To screen for Mmm antigens that induce IRG-47, we compared the genomes of the vaccine strain Ben-181 and the non-immunoprotective strain Ben-468, identifying 35 proteins absent in Ben-468 that may be associated with IRG-47 expression. Eukaryotic plasmids encoding these proteins were constructed and transfected into cells, leading to the identification of Mmm604, Mmm605, and Mmm606 as key IRG-47 inducers. Purification of these proteins with a His-tag further confirmed these findings. Intranasal immunization in mice demonstrated that rMmm604, rMmm605, and rMmm606 promote splenic lymphocyte proliferation, CD8 +T cell activation, and CD4 +T cell differentiation into Th1 cells, all of which are crucial for cellular immunity. Furthermore, all three proteins also stimulated the humoral immune response, while rMmm604 and rMmm606 induced the mucosal immune response. These findings suggest that rMmm604, rMmm605, and rMmm606 effectively induce cellular and humoral immune responses and are potential candidate antigens for Mmm subunit vaccine development. Our study highlights the potential of IRG-47 as a marker for screening candidate antigens for Mmm subunit vaccines.

## RESULTS

### Vaccine strain Ben-181 specifically upregulates host IRG-47 expression

**Fig 1:**
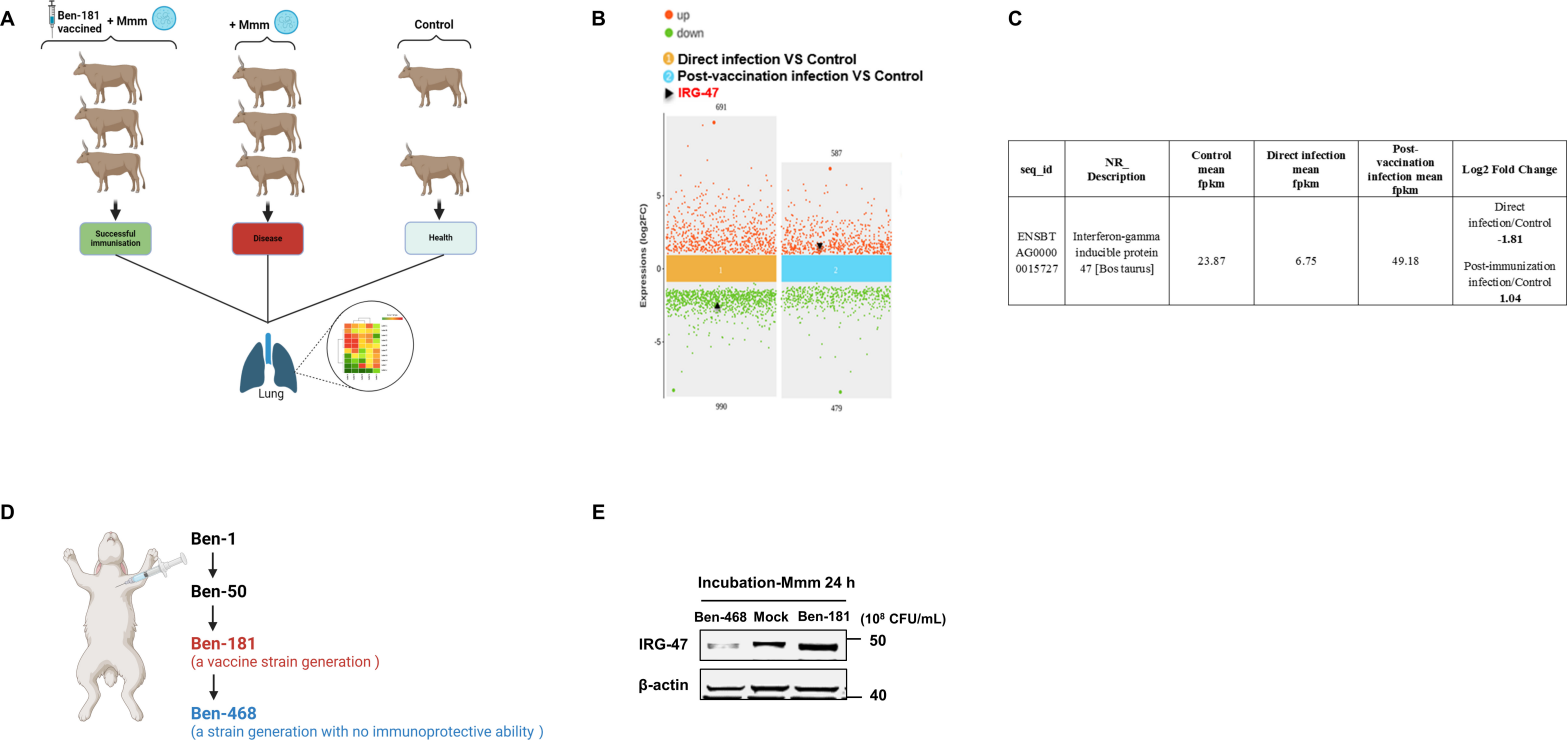
Vaccine strain Ben-181 specifically upregulates host IRG-47 expression. (**A**) Schematic representation of the bovine immune protection experiment involving the vaccine strain Ben-181. This work has not yet been published. (**B**) Scatter plot of differentially expressed genes comparing the groups Direct infection and Control (left panel) or Post-immunization infection and Control (right panel). The arrow indicates the location of IRG-47. (**C**) RNA-seq data of IRG-47 in the Direct infection, Post-immunization infection, and Control groups, with the ratio of Direct infection/Control or Post-immunization infection/Control in the Log2 format. (**D**) Representative passaged strains following Ben-1 inoculation into rabbits. (**E**) EBL cells were incubated with Ben-181 or Ben-468 for 24 h and then collected to assess IRG-47 protein levels by Western blotting analysis.

In a previous experiment on the immunoprotective effects of the Mmm vaccine strain Ben-181, we collected lung tissues from three groups of cattle: those vaccinated with Ben-181 and subsequently infected with the virulent strain Caprivi (no disease), those directly infected with the strain Caprivi (diseased), and a group of healthy controls. Transcriptome sequencing was performed on these samples (Fig. 1A). Comparative analysis revealed a significant upregulation of interferon-gamma (IFN-γ)-inducible protein 47 (IRG-47) in the lungs of the infected cattle post-vaccination. In contrast, IRG-47 expression was significantly lower in directly infected cattle compared to healthy controls (Fig. 1B and C). *In vivo* experiments suggested that the vaccine strain Ben-181 effectively induced IRG-47 expression. Consistent with *in vivo* findings, *in vitro* experiments demonstrated that Ben-181 upregulated IRG-47 expression in embryonic bovine lung epithelial (EBL) cells, whereas the non-immunoprotective strain Ben-468 did not (Fig. 1D and E).

Altogether, these results highlight that the Mmm vaccine strain Ben-181 specifically induces host IRG-47 release.

### Screening for Mmm antigens that induce host IRG-47 release

Our above experimental results demonstrate that the vaccine strain Ben-181 specifically induced host IRG-47 release, which is strictly regulated by IFN-γ release. Therefore, IRG-47 expression in response to Mmm antigens may serve as a marker for evaluating an antigen’s ability to stimulate IFN-γ release, facilitating antigen candidate screening. To test this, we first screened for Mmm antigens that trigger IRG-47 release and then verified their ability to elicit a cellular immune response.

**Fig 2:**
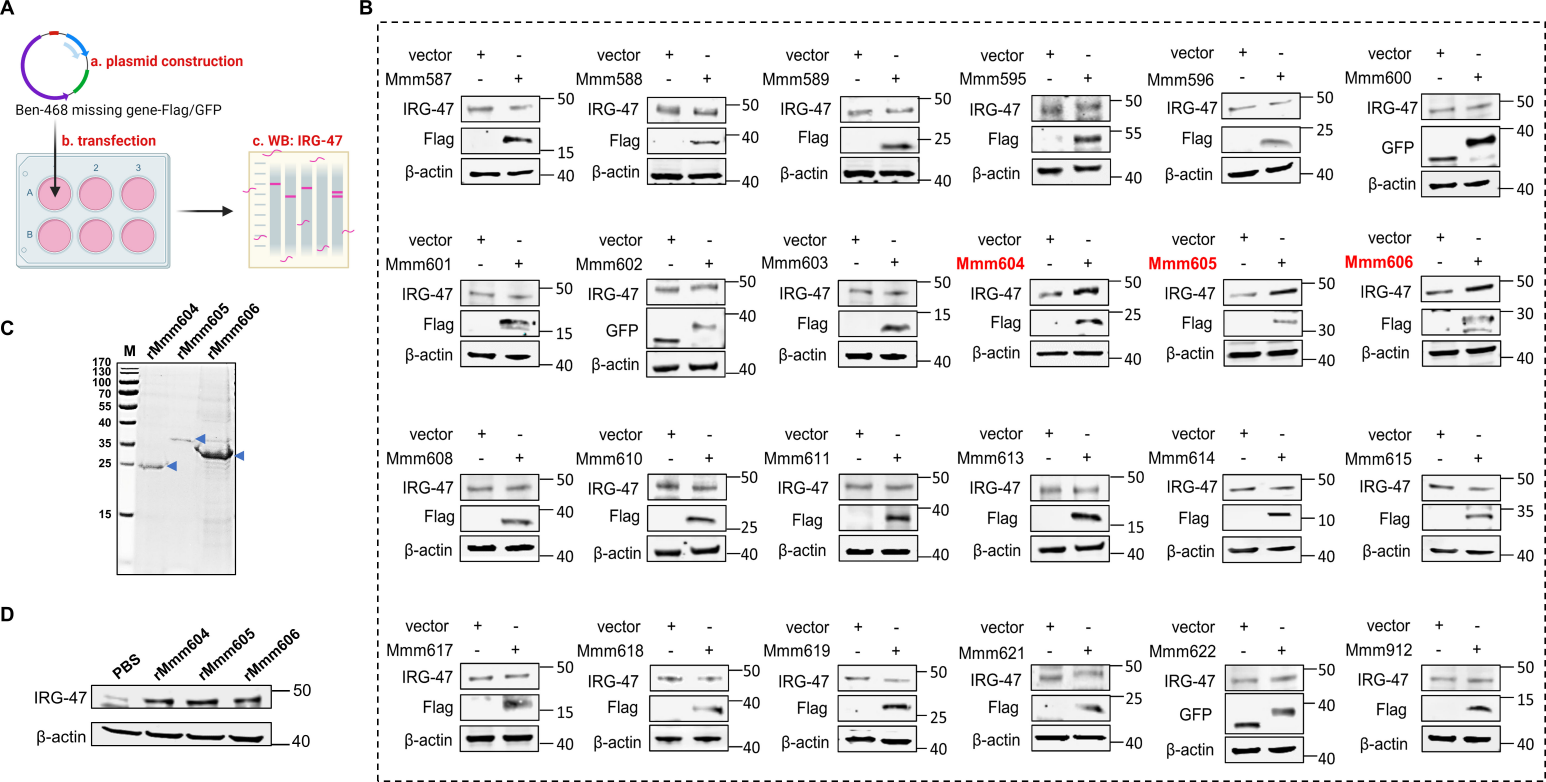
Screening for Mmm antigens that induce host IRG-47 release. (**A**) Diagram of the antigen screening process. (**B**) Flag- or GFP-tagged eukaryotic expression plasmid for each of the 35 proteins missing in Ben-468 was constructed and transfected into EBL cells. The cells were harvested to assess IRG-47 protein levels by Western blotting analysis. The same control sample (vector) was used across Mmm600 to Mmm602. The IRG-47 and β-actin blots for the vector sample were shared between Mmm600 and Mmm601, while the GFP blot for the vector sample was shared between Mmm600 and Mmm602. In addition, Mmm608 and Mmm611, Mmm614 and Mmm615, as well as Mmm622 and Mmm912 shared the same vector sample and its corresponding blots. (**C**) Purified His-tagged proteins rMmm604, rMmm605, and rMmm606 protein were identified by SDS-PAGE (the arrowhead). (**D**) EBL cells were incubated with 1 µg/mL rMmm604, rMmm605, or rMmm606 for 24 h and then collected to measure IRG-47 protein levels by Western blotting analysis.

Whole-genome sequencing of representative Ben strains revealed that Ben-468 had lost a genomic segment encoding approximately 35 proteins compared to the Ben-181 (18). Given that Ben-181 upregulated IRG-47 expression while Ben-468 did not, we hypothesized that these missing proteins might play a role in IRG-47 stimulation. To this end, we constructed eukaryotic expression plasmids with a Flag- or GFP-tag for each of the 35 proteins and transfected them into EBL cells. It was found that 24 proteins of these were successfully expressed and Mmm604, Mmm605, and Mmm606 significantly induced IRG-47 expression (Fig. 2A and B). We further purified these three proteins with a 6 × His tag and confirmed that recombinant proteins rMmm604, rMmm605, and rMmm606 stimulated host IRG-47 release (Fig. 2C and D). These findings indicate that we screened for Mmm antigens that induce IRG-47 expression—Mmm604, Mmm605, and Mmm606.

### rMmm604, rMmm605, and rMmm606 promote the proliferation and differentiation of mouse splenic lymphocytes

**Fig 3:**
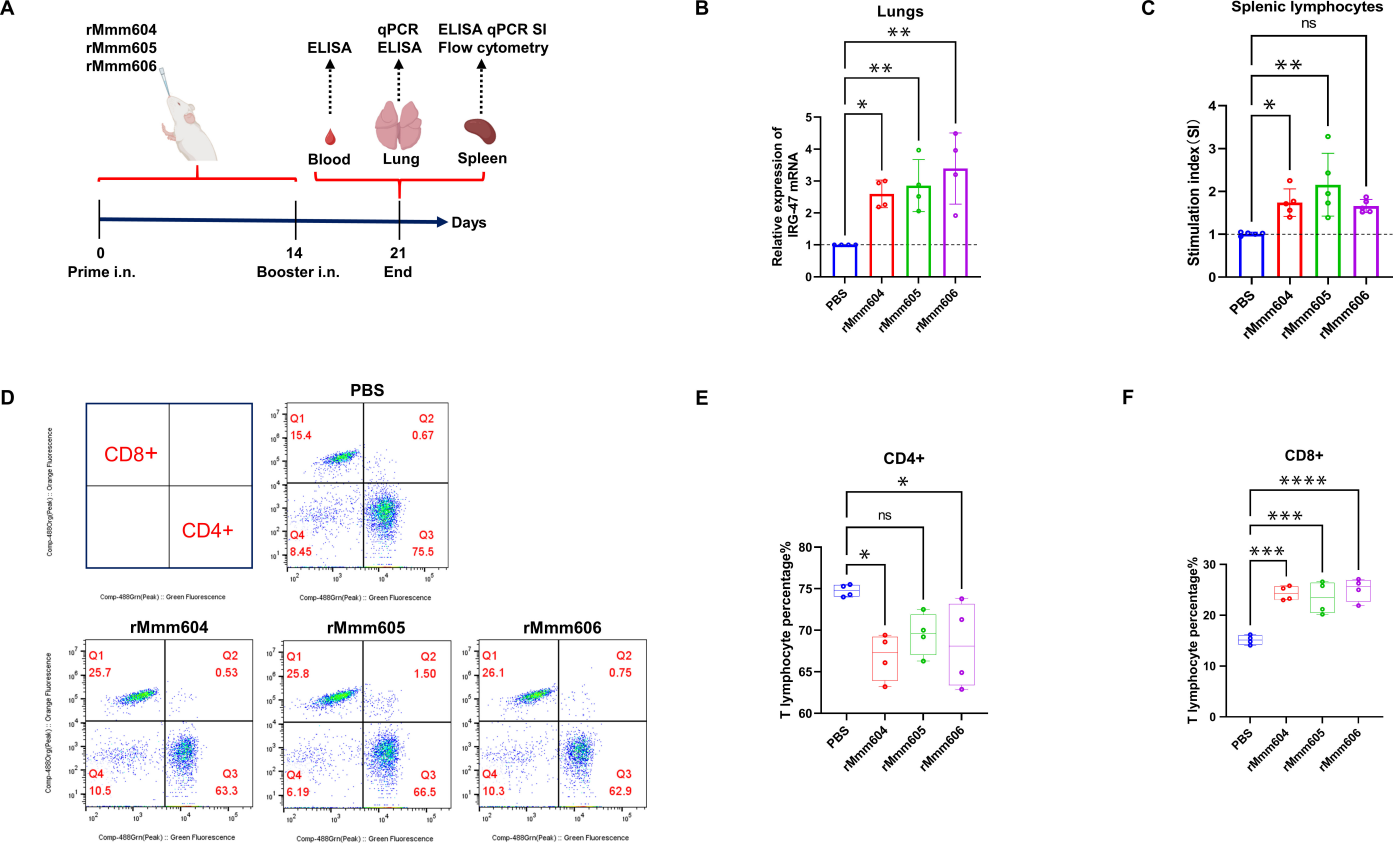
The recombinant proteins rMmm604, rMmm605, and rMmm606 promote the proliferation and differentiation of mouse splenic lymphocytes. (**A**) Schematic diagram of the immunization experiment in BALB/c mice. (**B**) Lung tissues from each group of mice were collected for IRG-47 transcription levels. (**C**) Lymphocyte proliferation assays in immunized mice. Splenic lymphocytes were isolated from each group and were further stimulated with the corresponding recombinant proteins. Lymphocyte proliferation was assessed using the CCK-8 assay. (**D**) Flow cytometry analysis depicting the percent of CD4 +CD8 T cells (bottom right) or CD4-CD8 + T cells (top left) in the mouse spleen. (**E and F**) Proportion of CD4 + and CD8+ T lymphocyte subsets in immunized mice spleen. The data are represented as the means ± SDs from at least three independent experiments and were normalized to the corresponding values in PBS (**B and C**). Significance was assessed by one-way ANOVA with Dunnett’s multiple comparison tests relative to the control. *, *P < 0.05*; **, *P < 0.01*; ***, *P < 0.001*; ****, *P < 0.0001*.

Next, we assessed the ability of rMmm604, rMmm605, and rMmm606 to stimulate cellular immune responses. BALB/c mice were intranasally immunized with each recombinant protein (Fig. 3A), and lung tissues were collected for qPCR analysis of IRG-47 expression. As shown in Fig. 3B, all three recombinant proteins promoted IRG-47 expression in mouse lung tissues.

Lymphocyte proliferation is the first step in the cellular immune response, creating effector lymphocytes to eliminate antigens (19). We thus isolated splenic lymphocytes from immunized mice and stimulated them *in vitro* with rMmm604, rMmm605, and rMmm606. The three proteins were observed to induce splenic lymphocyte proliferation (Fig. 3C), implying their role in eliciting a specific cellular immune response in mice.

To further investigate how rMmm604, rMmm605, and rMmm606 affect T lymphocyte differentiation to participate in the cellular immune response, we performed flow cytometry to analyze CD4 +T cells and CD8 +T cells subsets in the spleen of immunized mice. As illustrated in Fig. 3D through F, compared to the PBS-immunized control mice, mice immunized with the recombinant proteins exhibited a significant increase in CD8 +T cells and a corresponding decrease in CD4 +T cells. These findings indicate that rMmm604, rMmm605, and rMmm606 promote the differentiation of splenic T lymphocytes into the CD8 +subset, contributing to the cellular immune response.

Summarily, recombinant proteins rMmm604, rMmm605, and rMmm606 stimulate mouse splenic lymphocyte proliferation and the differentiation into CD8 +T cell, effectively enhancing the cellular immune response.

### rMmm604, rMmm605, and rMmm606 activate Th1 and Th2 responses

**Fig 4:**
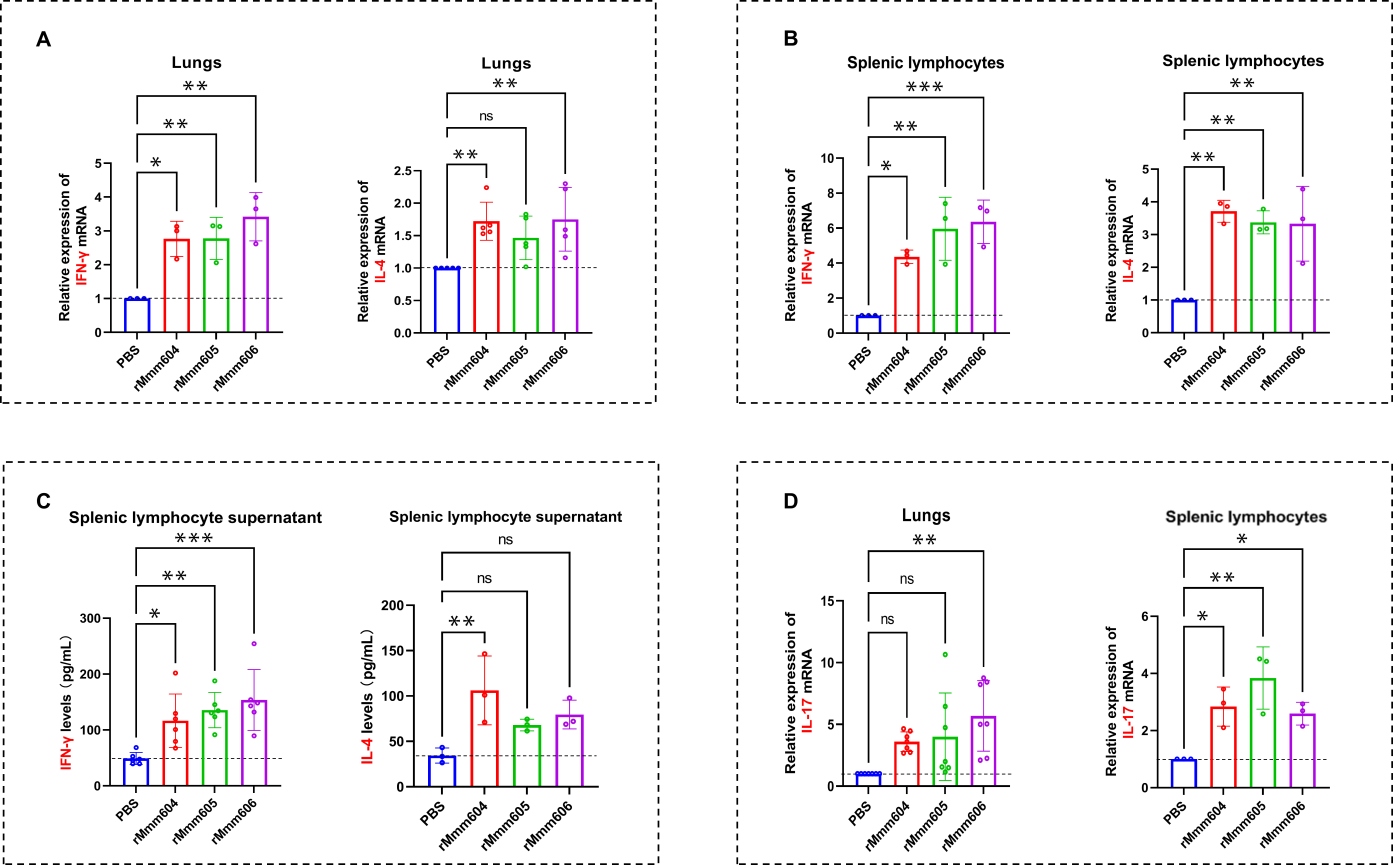
rMmm604, rMmm605, and rMmm606 activate Th1/Th2/Th17 responses. (**A**) Upregulation of IFN-γ and IL-4 expressions in the lung tissue of immunized mice was demonstrated by qPCR. (**B and C**) Splenic lymphocytes isolated from immunized mice and restimulated *in vitro* with the recombinant proteins. qPCR was used to evaluate IFN-γ and IL-4 mRNA levels in these splenic lymphocytes (**B**) and ELISA to analyze IFN-γ and IL-4 protein levels in their culture supernatants (**C**). (**D**) IL-17 mRNA levels in immunized mouse lung tissues and restimulated splenic lymphocytes were detected by qPCR. All data are represented as the means ± SDs from at least three independent experiments and were normalized to the corresponding values in PBS (**A, B, and D**). Significance was assessed by one-way ANOVA with Dunnett’s multiple comparison tests relative to the control. *, *P < 0.05*; **, *P < 0.01*; ***, *P < 0.001*.

To determine which response these three proteins tend to elicit, we examined the transcript levels of IFN-γ (a representative cytokine secreted by the Th1 subset) and IL-4 (a representative cytokine produced by the Th2 subset) in immunized mouse lung tissues. The qPCR analysis revealed that rMmm604, rMmm605, and rMmm606 upregulated IFN-γ and IL-4 mRNA expressions, but the increase in IFN-γ was higher than that of IL-4 (Fig. 4A), indicating the activation of both Th1- and Th2-type responses with a greater Th1 bias in mouse lung T lymphocytes. To further confirm these findings, the splenic lymphocytes were isolated from immunized mice and restimulated *in vitro* with rMmm604, rMmm605, and rMmm606. qPCR was used to evaluate IFN-γ and IL-4 mRNA levels in these splenic lymphocytes (Fig. 4B; Fig. S1) and ELISA to analyze IFN-γ and IL-4 protein levels in their culture supernatants (Fig. 4C). It was found that rMmm604, rMmm605, and rMmm606 significantly enhanced IFN-γ and IL-4 expressions at both the mRNA and protein levels in mouse splenic lymphocytes, with a greater upregulation of IFN-γ expression (Fig. 4B and C). These results further support their role in promoting Th1- and Th2-type responses, suggesting that rMmm604, rMmm605, and rMmm606 induce a Th1-biased or mixed Th1/Th2 immune response, contributing to both cellular and humoral immunity in mice, particularly cell-mediated immunity.

### rMmm604, rMmm605, and rMmm606 also trigger Th17 response

CD4 +T can also differentiate into the Th17 subset, which is characterized by IL-17 production and has been proposed to play a crucial role in pulmonary immune defense against respiratory pathogens (20). We thus performed a qPCR assay to assess IL-17 mRNA levels in immunized mouse lung tissues, as well as in mouse splenic lymphocytes restimulated with the recombinant proteins *in vitro*. As shown in Fig. 4D; Fig. S1, IL-17 mRNA levels were significantly boosted by all three proteins, implying that rMmm604, rMmm605, and rMmm606 also evoke a Th17 immune response in mice.

### The role of rMmm604, rMmm605, and rMmm606 in eliciting humoral and mucosal antibody responses

**Fig 5:**
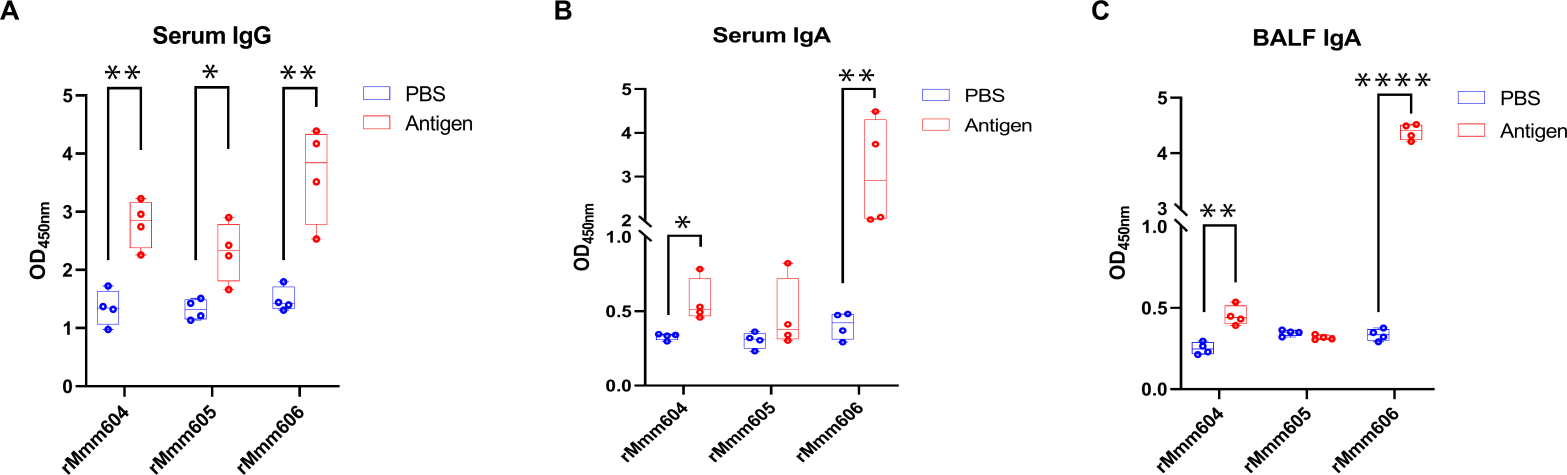
The role of rMmm604, rMmm605, and rMmm606 in eliciting humoral and mucosal antibody responses. (**A**) Serum-specific IgG antibodies of the mice immunized with rMmm604, rMmm605, and rMmm606 were determined by ELISA. (**B and C**) Specific IgA antibodies in immunized mouse serum (**B**) and BALF (**C**) were detected by ELISA. Data are presented as the means ± SDs of the results from four independent experiments. Significance was assessed by a two-tailed student’s *T* test. *, *P < 0.05*; **, *P < 0.01*; ****, *P < 0.0001*.

To evaluate the humoral immune response, we measured the serum antibody IgG levels in mice immunized with the three recombinant proteins using an indirect ELISA. At a 1:1,000 serum dilution, all three recombinant proteins exhibited OD 450 nm values greater than 2 and demonstrated a significant induction of specific IgG antibodies compared to the PBS-immunized control mice group (Fig. 5A). These data indicate that all three antigens effectively stimulate mouse humoral immune responses. Also, the mucosal immune response was analyzed by quantifying the specific IgA antibody levels in serum and BALF of immunized mice using an indirect ELISA. We observed that the levels of rMmm604- and rMmm606-specific IgA significantly increased compared to those of the PBS-immunized control mice group (Fig. 5B and C), suggesting that rMmm604 and rMmm606 exhibit a favorable role in stimulating mouse mucosal immunity.

Collectively, these results indicate that rMmm604, rMmm605, and rMmm606 induce humoral antibody responses, while rMmm604 and rMmm606 additionally stimulate mucosal antibody responses.

## DISCUSSION

A major challenge in subunit vaccine development is identifying antigens capable of eliciting a robust and comprehensive immune response (21). Current vaccine development strategies typically prioritize screening dominant antigens for humoral immunity, followed by assessing their ability to induce cellular and mucosal immune responses, and ultimately designing antigen combinations that elicit a comprehensive immune response (22–26). Given the vast number of *Mycoplasma* proteins, systematic antigen screening and rational combination remain significant challenges in subunit vaccine development. Recent studies indicate that high antibody levels induced by *Mycoplasma* vaccines do not directly correlate with immune protection, whereas cell-mediated immune responses play a more critical role in preventing *Mycoplasma*-induced infections (11, 27). Based on this understanding, the present study departed from traditional humoral immunity-based screening approaches and focused on identifying dominant Mmm antigens that elicit cellular immunity. We selected IFN-γ induction as the primary criterion for antigen screening, given that IFN-γ levels serve as a key indicator of cellular immune responses (28). It is not an uncommon strategy in subunit vaccine research, including for *Mycobacterium tuberculosis*. Intriguingly, based on the immunoprotection experiments with the successful CBPP-attenuated vaccine Ben-181, we observed that this vaccine specifically upregulates the expression of IRG-47, a protein strictly expressed under IFN-γ regulation. We speculated that a Western blotting detection of IRG-47 expression has the potential to be a more cost-effective and practical alternative to the commercial IFN-γ ELISpot assay for antigen screening. We subsequently initiated the screening process. Unlike Ben-181, Ben-468 failed to upregulate IRG-47 expression, suggesting that these 35 lost proteins in Ben-468 may be involved in IRG-47 upregulation and could serve as dominant antigens for inducing host cellular immunity. Of note, Ben-468 contained several gene insertions not found in Ben-181, raising the possibility that functional alterations in certain proteins contributed to the loss of IRG-47 upregulation. Future studies will seek to screen these candidate proteins to further elucidate their roles in immune activation.

We constructed eukaryotic expression plasmids encoding these 35 proteins, of which 24 were successfully expressed in EBL cells. Using the upregulation of host IRG-47 as a selection criterion, we identified three candidate antigens: Mmm604, Mmm605, and Mmm606. The three antigens are predicted to be localized on the cell membrane of Mmm. Mmm604 is an ABC-three-component system protein that is essential for bacterial growth, survival, and nutrient competition across diverse ecological niches (29, 30). Mmm605 is a Class II fructose-1,6-bisphosphate aldolase, a multifunctional enzyme involved not only in energy metabolism but also in plasminogen binding, transcriptional regulation, and host cell adhesion (31–33). Mmm606 belongs to the DeoR/GlpR family of DNA-binding transcriptional regulators, which typically function as global repressors of sugar metabolism (34). Notably, these three proteins are not unique to Mmm and are also present in some other mycoplasmas or bacteria, such as *M. leachii*, *M. feriruminatoris*, and *Streptococcus oralis.*

To evaluate their potential to activate the host immune response, we purified their recombinant proteins with a His-tag and administered them intranasally to mice. We first confirmed the ability of rMmm604, rMmm605, and rMmm606 to stimulate mouse splenic lymphocyte proliferation. Quantification of CD4 +and CD8+ T cells in the mouse spleen revealed that rMmm604, rMmm605, and rMmm606 promoted T lymphocyte differentiation into the CD8 +T cell subset, contributing to the cellular immune response. This may be effective at targeting intracellularly located Mmm. Increasing evidence suggests that *Mycoplasma* species can enter host cells to evade the immune responses and may lead to chronic infection, which may also explain the limited efficacy of humoral antibodies in preventing and controlling *Mycoplasma* infections (35, 36). Notably, a study on commercial vaccines against *M. hyopneumoniae* demonstrated an increased proportion of CD8 +T cells in vaccinated pigs. The authors suggest that CD8 +T cells play a protective role in *Mycoplasma* infections and could partially explain the beneficial effects observed after vaccination against *M. hyopneumoniae* (27, 37). CD4 +T cells play a central role in coordinating immune responses and can be classified into Th1, Th2, or Th17 subsets based on their cytokine profiles (38, 39). Th1 cells participate in cell-mediated immunity and stimulate B cells to produce opsonizing antibodies such as IgG2a, while Th2 cells contribute to humoral immunity by promoting the secretion of IgG1 and IgA (40). Th17 cells, which secrete IL-17, IL-17A, and IL-22, are particularly important for pulmonary immune defense against respiratory pathogens (41). IFN-γ, IL-4, and IL-17 are the effector cytokines produced by Th1, Th2, and Th17 cells, respectively. Analysis of their transcriptional and protein levels in immunized mouse lung tissues and splenic lymphocytes revealed that rMmm604, rMmm605, and rMmm606 induced a mixed Th1/Th2/Th17 immune response. A previous study on *M. pulmonis* infection using various T cell subset-depleted mice indicates that Th1, Th17, and CD8 +T cell responses are responsible for protection against *Mycoplasma* disease (12). The present study indicates that Mmm604, Mmm605, and Mmm606, identified based on host IRG-47 upregulation, effectively activate Th1, Th17, and CD8 +T cells, highlighting their potential as candidate subunit vaccine antigens.

Furthermore, we also evaluated the ability of rMmm604, rMmm605, and rMmm606 to elicit humoral and mucosal immune responses. All three proteins significantly induced a humoral immune response in mice, while rMmm604 and rMmm606 also stimulated a mucosal immune response. These findings suggest that rMmm604 and rMmm606 may serve as promising candidate antigens capable of inducing a comprehensive immune response in the host. Our current study represents a small-scale pilot investigation in mice. Future research will focus on optimizing the immunization protocols for the three proteins and conducting large-scale studies to further confirm their immunogenicity and efficacy. Future studies should also include immunization and protection assays in cattle to further assess their potential as Mmm subunit vaccine antigens.

In conclusion, our study presents a convenient and cost-effective approach for screening Mmm antigens that stimulate a host cellular immune response, given the crucial role of cellular immunity in preventing and controlling *Mycoplasma* infections. We hope this study may facilitate the identification of Mmm candidate antigens for the development of effective subunit vaccines.

## MATERIALS AND METHODS

### *Mycoplasma* strains, cells, and culture conditions

Mmm strain Ben-1 was isolated from the lung of a diseased cattle with pneumonia in Benxi of China. Ben-181 and Ben-468 were obtained by passage of Ben-1 in rabbits. These strains were identified by whole-genome sequencing and maintained in our laboratory (18). The Mmm strain Caprivi isolated from a CBPP-affected cow in the Namibian region of Caprivi (42) and provided by Dr. Geoffrey Muuka (Central Veterinary Research Institute, CVRI, Ministry of Fisheries and Livestock, Lusaka, Zambia). The Mmm strains were grown in PPLO medium and used at low passage number (< 10). To estimate the number of colony-forming units (CFUs) in the cultures, serial dilutions were plated on a modified PPLO medium containing 1.5% agarose (V2111; Promega) and incubated at 37°C. CFU was counted 7–10 days later using a microscope (43). The experiments with infectious Mmm were performed in the biosafety level 4 and animal biosafety level 4 facilities in the HVRI of CAAS.

Embryonic bovine lung epithelial (EBL) cells were kindly provided by Prof. Fei Xue of State Key Laboratory for Animal Disease Control and Prevention, Harbin Veterinary Research Institute (HVRI), Chinese Academy of Agricultural Sciences (CAAS), Harbin, China. The cells were cultured in Dulbecco’s modified Eagle’s medium (DMEM) supplemented with 10% heat-inactivated fetal bovine serum (FBS), 100 µg/mL streptomycin (Gibco), 100 U/mL penicillin (Gibco), and 10 mM HEPES (Invitrogen). The cells were incubated at 37°C in 5% CO_2_.

### Plasmid construction and transfection

The 35 nucleotide sequences missing from Ben-468 were codon-optimized and synthesized by Genesoul Technology (Harbin, China) and separately cloned into the pCAGGS-Flag vector with the restriction enzyme *EcoR* I and *Kpn* I or into the EGFP-C1 vector with the restriction enzyme *Sal* I and *Bam* HI. The PolyJet DNA *in vitro* transfection reagent (SignaGen, USA) was used to transfect recombinant plasmids into EBL cells.

### Western blotting

EBL cells were harvested at the indicated time points after different treatment strategies. An equal number of cells were lysed with the cell lysis buffer (50 mM Tris-HCl, pH 7.4, 150 mM NaCl, 1% Triton X-100, 2 mM EDTA, 0.1% SDS, 5 mM sodium orthovanadate) containing 1 × protease inhibitor cocktail (Roche Molecular Biochemicals) in an ice bath for 30 min. The protein concentration was determined using the BCA Protein Assay Kit (Beyotime, Nantong, China). Equal amounts of total cell lysates were separated by SDS-PAGE. The proteins in the gel were transferred onto nitrocellulose membranes (Cytiva), which were then blocked with 5% cold-water fish skin gelatin in TBST (20 mM Tris-HCl, pH 7.4, 150 mM NaCl, 0.1% Tween 20) for 1 h and then incubated for 2 h with the primary antibodies anti-IRG-47 (sc-390264, Santa Cruz), anti-Flag (F7425, Sigma-Aldrich), anti-GFP (50430-2-AP, Proteintech), or anti-β-actin (TA-09, Zhongshan Goldenbridge-Bio) at room temperature. After washing three times with TBST for 10 min each at room temperature, the membrane was incubated with 1: 10,000-diluted DyLight 800-labeled goat anti-mouse IgG (H + L) or DyLight 800-labeled goat anti-rabbit IgG (H + L) (1: 10,000, Kirkegaard & Perry Laboratories, Gaithersburg, USA) in TBST for 1 h at room temperature. The membrane was scanned in an Odyssey Infrared Imaging System (LI-COR Biosciences) after washing with TBST. The fluorescence intensity of each band was measured using Odyssey 2.1 software (LI-COR Biosciences).

### Protein expression and purification

The nucleotide sequence of Mmm604, Mmm605, and Mmm606 was cloned into the pET-28a vector with the restriction enzyme *Bam* HI and *Sac* I. The recombinant plasmid was transformed into *E. coli* BL21(DE3) cells. The level of recombinant protein expression was analyzed by SDS-PAGE. Subsequently, the 6 × His-tagged recombinant proteins rMmm604, rMmm605, and rMmm606 were purified by using Ni Sepharose 6FF (Cytiva) according to the manufacturer’s instructions. These proteins were concentrated and resuspended in PBS using a 10 kDa ultrafiltration tube (Millipore). The protein purification was analyzed by SDS-PAGE, and the protein concentration was determined with a BCA Protein Quantitation Kit (Beyotime, Shanghai, China).

### Immunization experiments

The 40 six-week-old female specific-pathogen-free (SPF) BALB/c mice used in the experiments were obtained from Beijing Vital River Experimental Animals Technology (Beijing, China). The animals were randomly divided into four groups. A sample size of 10 mice per group was selected to ensure robust statistical analysis while adhering to the 3R principles of animal welfare (Replacement, Refinement, and Reduction), as well as considering precedents from similar published studies (44–47). Groups 1–3 received 20 µg of the purified proteins rMmm604, rMmm605, or rMmm606 mixed with an equal amount of CpG ODN 2395 (Biodragon, Suzhou, China) via intranasal inoculation (i.n.), respectively. Group 4 received the same volume of PBS containing 20 µg CpG ODN 2395 as the negative control. Each animal was boosted with the same dose 14 days after the first inoculation.

### Sample collection

Serum samples were obtained from the retro-orbital sinus before immunization and at 21 days after the first inoculation. The mice were then humanely euthanized and necropsied, and BALF samples were collected from four mice per group. Then, 1  mL sterile PBS (pH 7.2) was slowly infused into the lungs and passaged for 30  seconds. The recovered fluid was immediately cooled at 4°C and centrifuged at 300 × *g* for 10  min. The serum samples, lung tissue, spleen tissue, and BALF samples were stored at −80°C for later use.

### Specific lymphocyte proliferation response assay

Splenic lymphocytes were isolated from mouse spleens with mouse lymphocyte separation medium (Dakewe, Beijing, China) under sterile conditions. The splenic lymphocyte suspension was then resuspended in complete culture medium at a concentration of 5 × 10^6^ cells/mL. The cells were seeded into 96-well plates and incubated at 37°C in a humidified incubator with 5% CO_2_. The cells were stimulated with the corresponding purified proteins rMmm604, rMmm605, and rMmm606 at a final concentration of 10 µg/mL or PBS at 37°C for 48 h. After incubation, 10 µL of CCK-8 solution was added to each well without discarding the culture medium, and the cells were cultured for another 4 h. The OD values were measured at 450  nm (OD450) with a spectrophotometer plate reader (BioTek). The stimulation index (SI) values were calculated as the ratio of the OD450 of the antigen-stimulated wells to that of the unstimulated wells.

### Flow cytometric determination of splenic lymphocyte subpopulations

Splenic lymphocytes were incubated with the anti-mouse CD3+ (T cells) antibody conjugated to fluorescein allophycocyanin (APC) (Biolegend), anti-mouse CD4 +antibody conjugated to isothiocyanate (FITC) (Biolegend), and anti-mouse CD8 +antibody conjugated to phycoerythrin (PE) (BD Pharmagen) for 30  minutes at 4°C. The labeled cells were then washed and resuspended in PBS at 250 × *g* for 10  minutes for further analysis using flow cytometry (A60-Universal, Apogee, Britain). All flow cytometry data were analyzed using the FlowJo software (version X10.0; FlowJo, LLC, Ashland, OR).

### Cytokine measurement in mouse lung tissue, splenic lymphocytes, and culture supernatants of splenic lymphocytes

The mouse splenocytes were cultured in 24-well plates at a concentration of 5 × 10^5^ cells/well. Cell suspensions were incubated at 37°C in 5% CO_2_ for 72 h with the corresponding purified rMmm604, rMmm605, and rMmm606 individually at a final concentration of 10 µg/mL. The supernatant was harvested and clarified by centrifugation to remove the cell debris. The levels of IFN-γ and IL-4 were measured with corresponding ELISA kits (Dakewe, Beijing, China).

Mouse spleen cells and lung tissues were collected to extract the total RNA with a Total RNA Extraction Kit (SEVEN, Beijing, China). The total RNA was reverse-transcribed with a cDNA synthesis kit (Vayzme, Nanjing, China), and then the mRNA levels of IFN-γ, IL-4, and IL-17 were assessed by quantitative real-time PCR (RT-qPCR). RT-qPCR was performed in triplicate using QK Platinum SYBR Green Master Mix (Thermo Fisher). All data were acquired using a QuantStudio 5 real-time PCR system (Applied Biosystems, Carlsbad, USA). The expression value of each gene was normalized to that of β-actin, and final values were calculated using the ΔΔCt method. The results were analyzed using QuantStudio Design & Analysis software v1.4 (Applied Biosystems). The primer sequences used in this study are listed in Table 1.

**TABLE 1:** Primers used in this study

Primer name	Sequence (5’−3’)
Mmm604-F^*a*^	CGGGATCCATGACCCAGAAGAACATCAA
Mmm604-R^*a*^	CGAGCTCGATGATCTCCACCTCCTTG
Mmm605-F^*a*^	CGGGATCCATGAAGGCCAGCCTGAAG
Mmm605-R^*a*^	CGAGCTCGGCCTTGTTGATGCTGC
Mmm606-F^*a*^	CGGGATCCATGTTAAAAGATCAAAGAA
Mmm606-R^*a*^	CGAGCTCTTCAAGGTTTATAATTAGTC
IFN-γ-F^*b*^	TCAAGTGGCATAGATGTGGAAGAA
IFN-γ-R^*b*^	TGGCTCTGCAGGATTTTCATG
IL-4-F^*b*^	GAGCTGCAGAGACTCTTTCG
IL-4-R^*b*^	CATGGTGGCTCAGTACTACG
IL-17-F^*b*^	CGCAATGAAGACCCTGATAGAT
IL-17-R^*b*^	CTCTTGCTGGATGAGAACAGAA
β-actin-F^*b*^	GCAGGAGTACGATGAGTCCG
β-actin-R^*b*^	ACGCAGCTCAGTAACAGTCC

^*a*^ These primers are employed to generate recombinant plasmids with T4 DNA ligase, and restriction enzyme sites incorporated into primers are underlined.

^*b*^ The primers are used in quantitative PCR (qPCR).

### Determination of specific IgG and IgA responses elicited by the recombinant proteins using ELISA

The 96-well plates were coated with 100 µL of 5 µg/mL rMmm604, rMmm605, or rMmm606 proteins in each well and incubated at 4°C overnight. The wells were then washed three times with PBST and blocked with 200 µL/well PBS containing 5% (wt/vol) cold-water fish skin gelatin at 37°C for 2 h. After the wells were washed three times with PBST, 100  µL of serum (diluted 1:1000 in PBS) or BALF (diluted 1:10 in PBS) corresponding to the antigen was added to the respective wells and incubated at 37°C for 1  h. Subsequently, the wells were washed three times with PBST and incubated with 100  µL/well of anti-mouse IgG antibodies (Zhongshan Goldenbridge-Bio) (diluted 1:5,000 in PBST), or with anti-mouse IgA antibodies (Abcam) (diluted 1:10,000 in PBST), both conjugated with HRP, at 37°C for 1  hour. After three washes, 100  µL of 3,3’−5,5’-tetramethyl benzidine substrate (TMB) was added to each well. Following a 15 min incubation away from light, the reaction was halted by adding 100  µL of 2  mol/L H_2_SO_4_, and the absorbance was measured at 450  nm using a spectrophotometer plate reader (BioTek).

### Statistical analysis and visualization

GraphPad Prism software (version 9.0; GraphPad Software Inc.) was used for all statistical analyses. Data obtained from at least three independent experiments are reported as mean  ±  SD. The number of replicates for each experiment was determined using G*Power 3.1 software (48), with the parameters set at α = 0.05 and a power ≥0.8. The significance of differences between the two groups was determined with a two-tailed Student’s *t* test. One-way analysis of variance (ANOVA) with Dunnett’s multiple comparison tests relative to the control was employed for multigroup comparisons. For all analyses, a probability (*P*) value of <0.05 was considered statistically significant. Schematic diagrams or elements in some figures were drawn using BioRender (https://app.biorender.com/).

## SUPPLEMENTAL MATERIAL

Fig. S1 iai.00186-25-s0001.tif External positive controls.

ASM does not own the copyrights to Supplemental Material that may be linked to, or accessed through, an article. The authors have granted ASM a non-exclusive, world-wide license to publish the Supplemental Material files. Please contact the corresponding author directly for reuse.
