# Supplementary figures and images for: Evaluation of Mycoplasma mycoides subsp. mycoides antigens capable of stimulating host IRG-47 release identifies Mmm604, Mmm605, and Mmm606 as potential subunit vaccine antigens
Source: Infect Immun. 2025 Sep 9;93(10):e00186-25. doi: 10.1128/iai.00186-25 (PMC12519791; doi:10.1128/iai.00186-25)

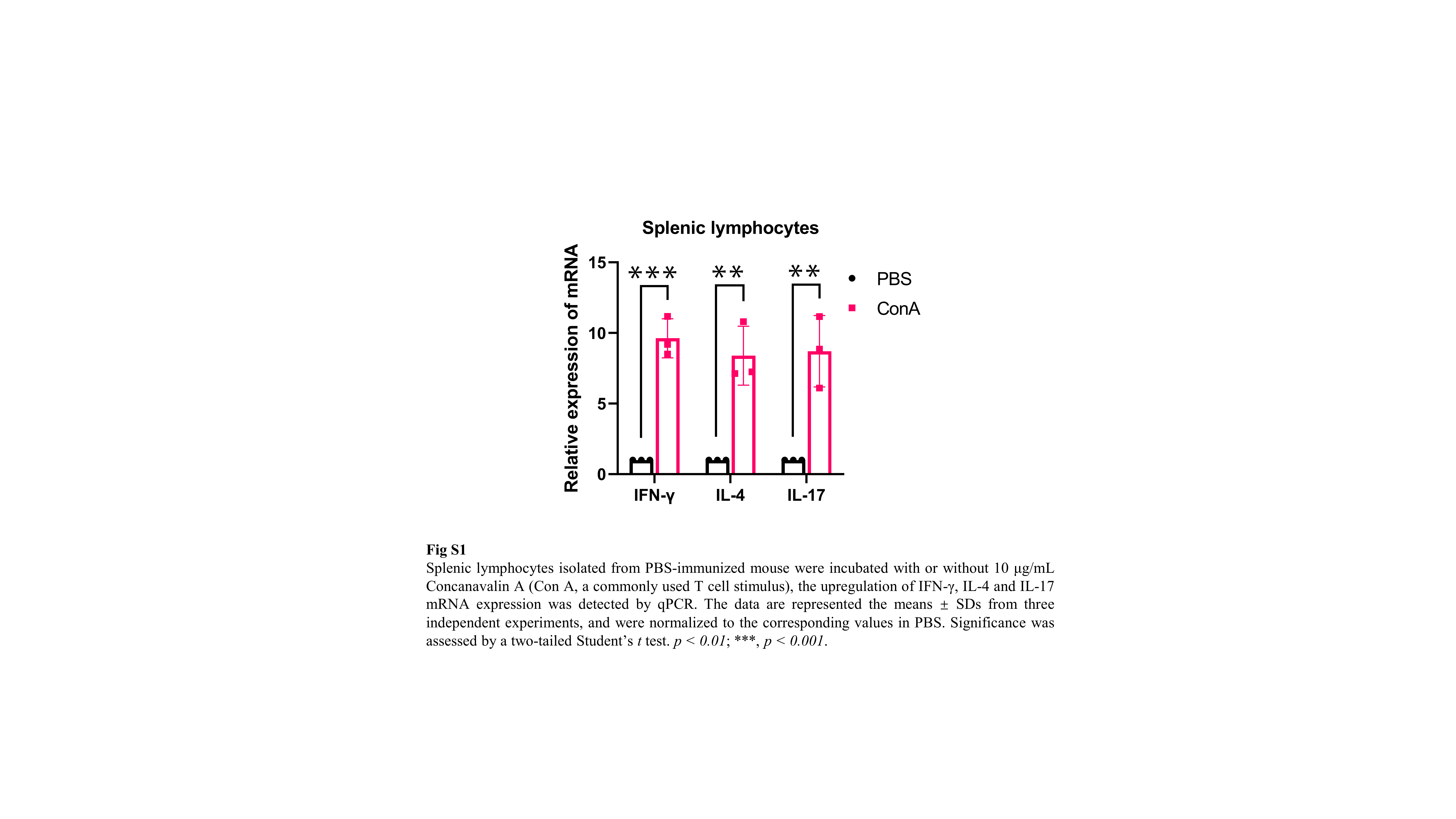

Supplement: Fig. S1 — External positive controls. [file iai.00186-25-s0001.tif]
